# The Tumor-Associated Marker, PVRL4 (Nectin-4), is the Epithelial Receptor for Morbilliviruses

**DOI:** 10.3390/v6062268

**Published:** 2014-06-02

**Authors:** Sebastien Delpeut, Ryan S. Noyce, Christopher D. Richardson

**Affiliations:** 1 The Department of Microbiology and Immunology, Dalhousie University, Halifax, B3H 1X5 NS, Canada; E-Mails: sebastien.delpeut@dal.ca (S.D.); noycer@dal.ca (R.S.N.); 2 IWK Health Centre, Canadian Center for Vaccinology, Goldbloom Pavilion, Halifax, B3H 1X5 NS, Canada; 3 The Department of Pediatrics, Dalhousie University, Halifax, B3K 6R8 NS, Canada

**Keywords:** morbillivirus, measles virus, canine distemper virus, peste des petits ruminants virus, nectin-4, PVRL4, epithelial receptor, cancer

## Abstract

PVRL4 (nectin-4) was recently identified as the epithelial receptor for members of the *Morbillivirus* genus, including measles virus, canine distemper virus and peste des petits ruminants virus. Here, we describe the role of PVRL4 in morbillivirus pathogenesis and its promising use in cancer therapies. This discovery establishes a new paradigm for the spread of virus from lymphocytes to airway epithelial cells and its subsequent release into the environment. Measles virus vaccine strains have emerged as a promising oncolytic platform for cancer therapy in the last ten years. Given that PVRL4 is a well-known tumor-associated marker for several adenocarcinoma (lung, breast and ovary), the measles virus could potentially be used to specifically target, infect and destroy cancers expressing PVRL4.

## 1. Introduction

Morbilliviruses, which include measles virus (MeV), canine distemper virus (CDV) and peste des petits ruminants virus (PPRV), are among the most widespread and highly contagious pathogens in their respective hosts. Lapses in vaccination have produced frequent outbreaks of measles in recent years. Although measles virus infects human and non-human primates, humans are the only reservoir for MeV [1]. On the other hand, CDV, isolated from dogs, infects a wide range of wild carnivore species [2,3,4], while PPRV primarily infects small ruminants, such as sheep and goats [5]. The role of wildlife as reservoir hosts for PPRV remains unclear [5]. The genus, *Morbillivirus*, belongs to the Paramyxoviridae family in the order, Mononegavirales. The genome is a negative-sense, non-segmented, single-stranded RNA, and its structure is conserved with the nucleocapsid (N), phosphoprotein (P), matrix (M), fusion (F), attachment (H) and large (L) genes present in all morbilliviruses [6]. MeV, CDV and PPRV are not only genetically related, but also induce similar symptoms in their hosts, such as fever, respiratory and gastrointestinal signs, often complicated by secondary bacterial infections, due to morbillivirus-induced immunosuppression [7,8,9,10]. Interestingly, neurological disease caused by MeV is rare, whereas with CDV, it is more frequent. Central nervous system complications are not associated with PPRV infection [11]. As well as producing an enormous health burden on human, livestock and wildlife health by causing mild to severe or fatal diseases, the economic impact of these human and animal viruses is considerable [3, 12,13,14,15,16,17]. 

This review highlights recent advances on the role of PVRL4 (nectin-4) during morbillivirus infections. Specifically, the function of PVRL4 during morbillivirus entry and exit, and, in particular, the role the variable (V) domain of PVRL4 plays in the recognition of morbillivirus attachment receptors, is discussed. Finally, the oncolytic potential of morbilliviruses in targeting PVRL4 as a tumor marker on adenocarcinomas is proposed. 

## 2. Routes of Morbillivirus Entry

**Figure 1:**
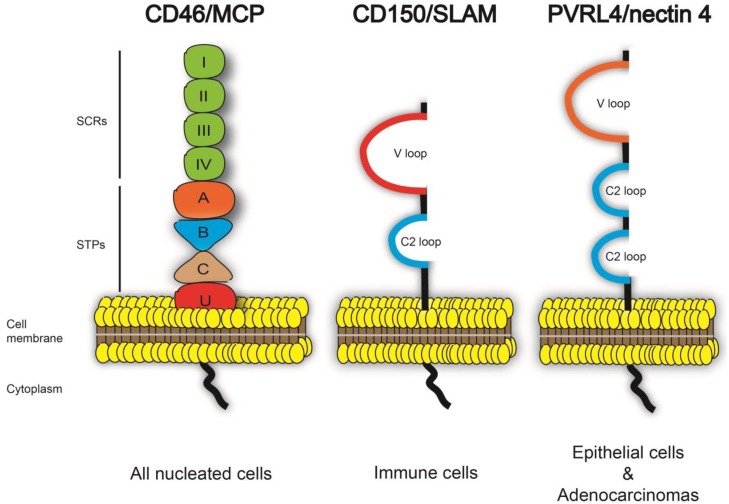
Structure of *Morbillivirus* receptors. (**A**) CD46/MCP structure consists of four short consensus repeats (SCRs I, II, III and IV), a serine/threonine/proline region (STPs A, B and C), a sequence of unknown significance (U), a transmembrane sequence and a cytoplasmic domain. Only vaccine or laboratory adapted strains of measles virus (MeV) use CD46 as a receptor. (**B**) CD150/SLAM contains a variable (V) domain and a constant (C2) Ig-like repeat in its extracellular domain. SLAM is the universal immune receptor for all morbilliviruses. (**C**) The PVRL4/nectin-4 extracellular domain is composed of a variable domain and two C2 domains. To date, PVRL4 was shown to serve as an epithelial receptor for measles virus, canine distemper virus and peste des petits ruminants virus.

**Figure 2:**
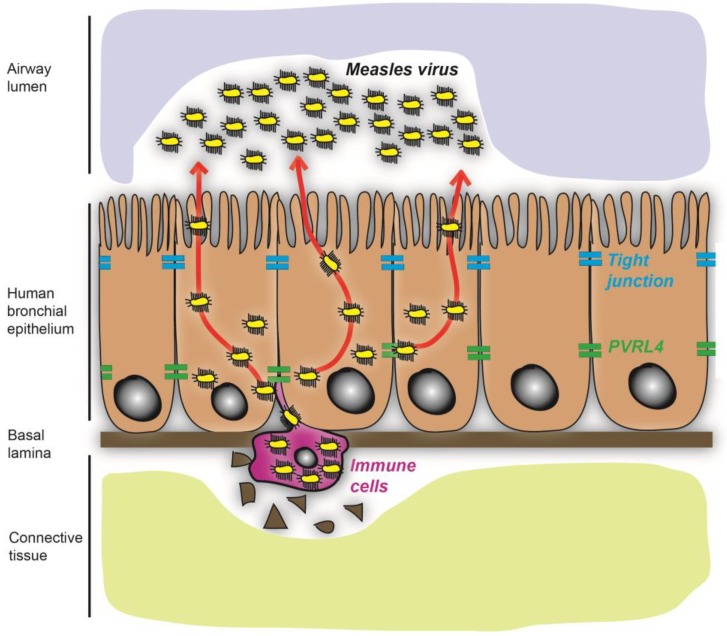
The entry and release of measles virus in human bronchial epithelium. MeV infects the basolateral surface of well-differentiated human airway epithelia via infected immune cells, which migrate to and interact with the adherens junction molecule, PVRL4. The infection subsequently spreads to adjacent cells via PVRL4. Later, measles virus is released from the apical surface of ciliated cells into the airway lumen.

Most morbilliviruses have established lymphotropism and epitheliotropism that are receptor-dependent (Figure 1) [18,19,20,21,22,23,24,25]. Interestingly, vaccine and laboratory-adapted strains of MeV can use the human membrane cofactor protein, CD46/MCP, as an additional receptor, resulting from the adaptation of MeV to growth in cell culture [26, 27]. However, CD46/MCP is not a natural receptor involved in wtMeV pathogenesis and is not used by other morbillivirus species. Specific interactions between cellular receptors and the viral hemagglutinin protein (H) facilitate virus entry into host cells [18,19,20,21,22,23,24,25] by inducing virus-cell and cell-cell membrane fusion in cooperation with the fusion protein (F) [28,29,30,31]. Importantly, morbilliviruses are highly lymphotropic viruses that use the signaling lymphocyte activation molecule (SLAM/CD150) as an immune cell entry receptor that is expressed on the surface of activated T- and B-lymphocytes, macrophages and dendritic cells [23,24,25, 32,33,34]. During infection, tissue resident dendritic cells and macrophages initially become infected and migrate to the lymph nodes, where the virus has access to a large pool of activated T- and B-lymphocytes [6, 35, 36]. These lymphocytes disseminate the virus throughout the lymphatic system. The appearance of clinical signs coincides with viral spread to epithelial cells [1, 33, 37]. Finally, infected cells and virus subsequently shed in respiratory secretions, urine and feces [38,39,40,41,42,43]. Ruminants, carnivores or primates can become infected through close contact with or eating sick prey [40, 44,45,46,47] (Figure 2). While SLAM is not expressed in epithelial tissues, PVRL4 (nectin-4) was recently identified as the epithelial receptor for MeV, CDV and PPRV [18,19,20,21,22]. 

### 2.1. PVRL4 Expression and Distribution

Expression and distribution of cellular morbillivirus receptors in a host is particularly important, because the interaction of a virus with its cellular receptor links virus tropism and pathogenesis [48]. High levels of ovine PVRL4 transcripts are found in epithelial tissues, including the mouth, the upper respiratory tract and the stomach [19], while human PVRL4 is abundantly expressed in placental trophoblasts, gastric glandular cells and adenocarcinomas of the lung, breast and ovary [49]. In the human respiratory tract, PVRL4 expression is a prerequisite for MeV infection of both well-differentiated human airway epithelial cells [22] and primary human small airway epithelial cells grown in the presence of serum [21]. Interestingly, PVRL4 is expressed on both the apical and basolateral surfaces of a number of polarized adenocarcinoma cells, where MeV infection is more efficient via the apical route [21]. Direct virus binding and entry via the apical surface of normal airway epithelial cells does not appear to occur during initial virus exposure. Indeed, it was previously reported that ciliated epithelial cells are refractory to MeV infection from the apical side, preventing them from being the primary target cells [50, 51]. In contrast, MeV preferentially infects differentiated primary epithelial cells via the basolateral route, which is consistent with polarized expression of PVRL4 along the basolateral surface of pseudostratified ciliated epithelial cells in the trachea and nasal concha [51,52,53,54]. PVRL4 actually plays a key role in virus spread from immune to epithelial cells [54,55,56]. A study with monkeys highlights the fact that MeV preferentially infects the basolateral surface of well-differentiated human airway epithelia via the migration of CD150-expressing myeloid cells into the tracheal epithelium [55]. These findings were corroborated by another study showing that MeV infection of epithelial cells in the macaque upper respiratory tract is mediated by subepithelial CD150 positive B-lymphocytes [54]. Once MeV gains entry into the respiratory tract, the spread of the virus is primarily mediated by PVRL4. Although wtMeV spreads efficiently to the epithelium in tracheal infections compared to PVRL4-blind MeV infection in monkeys [55], the lack of an epithelial infection by PVRL4-blind MeV could result from the more rapid clearance of this virus *in vivo* [55]. Moreover, MeV is released from the apical membrane of polarized epithelia [55, 57,58,59,60]. Rhesus macaques infected with epithelial receptor-blind MeV developed clinical symptoms similar to monkeys infected with wild-type MeV, but the virus was unable to cross the airway epithelium to be shed in the airways [52]. Similarly, the epithelial receptor-blind CDV was not detected in epithelial tissues from infected ferrets [37]. Taken together, these data suggest a novel model for the spread of morbilliviruses through the respiratory tract epithelia, which requires PVRL4 as an exit receptor. 

Nevertheless, morbillivirus pathogenesis cannot be explained by the presence of SLAM and PVRL4 alone. Small ruminants infected with PPRV often exhibit lesions in the intestine that express relatively low levels of SLAM and PVRL4 [19, 61, 62]. In contrast, higher levels of cytoplasmic PVRL4 expression in the stomach epithelial cells [49] does not correlate with the gastric pathology associated with PPRV infection [19, 61, 62]. As a consequence of these observations, the relevance of PVRL4 accessibility and localization within different epithelial cells should also be considered, as this might influence virus spread beyond just PVRL4 expression levels. Additional unidentified cellular receptors may also play a key role in morbillivirus-induced pathogenesis within the central nervous system, as complications associated with MeV and CDV infections can arise here at late stages of the disease. Finally, virus replication might also be blocked by a restriction factor in cells permissive to morbillivirus entry [63].

## 3. Structural Insights into the Interaction of Morbilliviruses with PVRL4

The characterization of virus-receptor interactions is particularly important to better understand virus entry and offers new opportunities for developing antiviral therapies. PVRL4 is a member of the nectin family of adhesion molecules, which belongs to the immunoglobulin (Ig) superfamily, comprised of nectin-1, -2, -3, -4 and the prototypic poliovirus receptor (PVR) [64,65,66]. PVR mediates the entry for poliovirus [67], while PVRL1 and 2 serve as a receptor for herpes simplex viruses [68]. Nectins are normally localized to the adherens junctions and are components of the cell-cell adhesion system, where they play a key role in limiting cell movement, facilitating intercellular communication and regulating proliferation [64,65,66]. PVRL4 is a type I transmembrane glycoprotein with three Ig-like ectodomains (V and two C2 domains), a transmembrane region and a cytoplasmic tail [65, 69]. V domains are involved in homotypic or heterotypic interactions with nectin-1, while C2 domains enhance the affinity of these interactions [66, 69, 70]. PVRL4 initially interacts with other PVRL4 molecules in *cis-*, followed by *trans*-interactions with PVRL4 on adjacent cells, while PVRL4-PVRL1 interactions only appear *in trans* on adjacent cells [69, 71,72,73]. As a consequence of other binding partners for PVRL4, it will be of interest to determine if such PVRL protein interactions might influence morbillivirus entry. 

### 3.1. The V Domain of PVRL4 is Essential for Morbillivirus Entry

**Figure 3:**
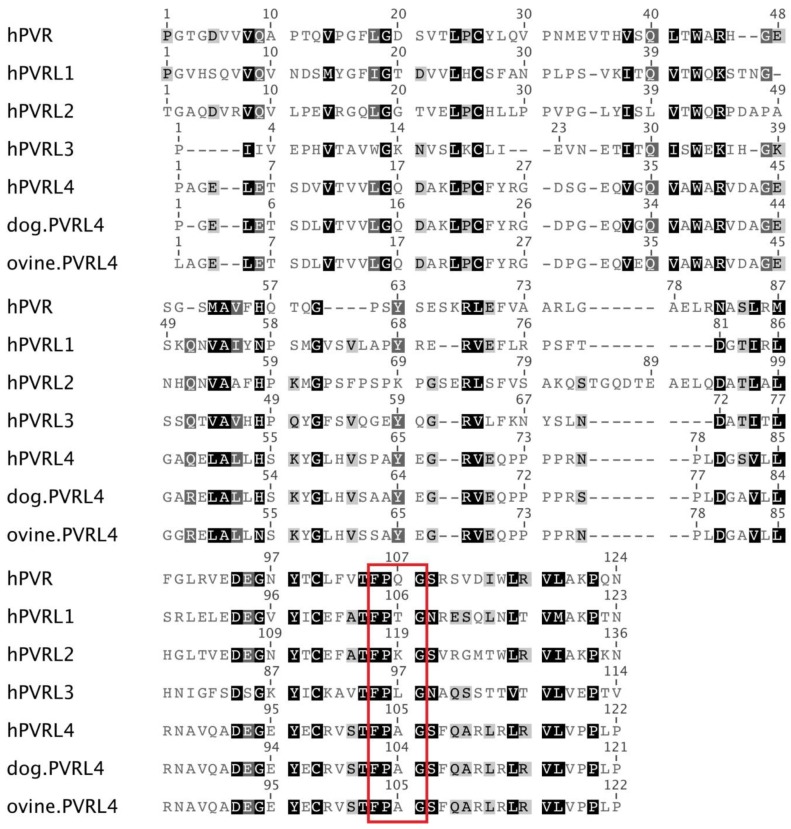
Amino acid sequence alignment of the V domains from human PVRL1 to PVRL4, human PVR, dog PVRL4 and ovine PVRL4. Residues having similarity are shaded (dark shading represents identical amino acid residues; dark grey shading represents residues with 80%–100% identity, light grey shading represents residues with 60%–80% identity, no shading represents residues with less than 60% identity). The consensus FPxG amino acid sequence is boxed in red. The Geneious sequence alignment software was used to perform pairwise protein alignments [78].

PVRL4 was biochemically shown to support binding to MeV H through its V domain, leading to virus entry [22]. This is similar to the scenario where the V domain of SLAM also interacts with MeV H [74]. Interestingly PVRL4-MeV H interactions are stronger than MeV H binding to SLAM, although PVRL4-MeV-induced syncytia formation is less extensive compared to SLAM [22]. In cell culture, the PVRL4 V domain was shown to be essential for CDV infection using whole chimeric nectins, where the V domains of PVRL4 and PVRL1 were exchanged [75]. A crystallographic structure of the ligand-binding domains between PVRL4 and MeV H has identified three binding interfaces [76]. Sites II and III are respectively located in the B-C and C’-C’’ loops of the PVRL4 V domain and contribute to receptor-ligand interactions [76]. In contrast, the F-G loop containing Site I interacts with two hydrophobic patches in MeV H, conferring strong stabilizing forces [76]. Importantly, mutations F101S, P102S, A103S and G104Y in Site I of the PVRL4 V domain almost completely abolished MeV H recognition *in vitro* [76]. This FPAG motif (F101, P102, A103 and G104) within the V domain of PVRL4 is conserved in the related PVRL4 V domains from ovine and dog species [18,19,76] (Figure 3) and is critical for CDV entry and virus spread [75]. Although FPxG is a consensus amino acid sequence located in all human nectin V domains [76, 77], PVRL1, PVRL2 and PVRL3 are not used as a receptor by MeV [21, 22]. Taken together, these studies demonstrate that morbillivirus epithelial cell infections are highly conserved, since both MeV and CDV H proteins share key PVRL4 binding residues [37, 52, 75]. 

### 3.2. High Similarity between the PVRL4 Receptors from Different Origins Might Weaken the Species Barrier for Morbillivirus

In contrast to MeV and PPRV, canine distemper virus infects a wide range of hosts, highlighting its ability to jump across species. Interestingly, PVRL4 sequences from human and dog origin are almost identical at the amino acid level, and both V domains contain the FPAG motif [18, 19]. Due to these similarities, CDV has the intrinsic ability to use both human and dog PVRL4 as an epithelial receptor, without a requirement for adaptive mutations in H [79, 80]. Thus, CDV can replicate in human epithelial cells after blocking the host innate immune response [81]. Although a single mutation in the hemagglutinin gene is still required for CDV to use the human SLAM entry receptor [79, 80], canine distemper virus has the potential to emerge as a novel human pathogen [80]. Indeed, natural infections with CDV have already been reported in non-human primates [46, 47], including an outbreak of CDV in rhesus monkeys at a breeding farm in China [45]. However, a recent study suggests that additional mutations are likely necessary to achieve full virulence in non-natural hosts [82]. On the other hand, the chance of CDV adapting to humans is low, due to the presence of cross-reactive cellular and humoral immunity against pre-existing MeV antigens induced by the MMR vaccine or childhood infection [83,84,85]. A study performed with monkeys proved that measles vaccination can prevent CDV from crossing species barriers to infect primates [82]. Importantly, these studies imply that measles eradication and lapses in MMR vaccination could allow CDV to emerge and infect humans. 

## 4. The Oncolytic Potential of Measles Virus

During the past decade, measles virus vaccine strains have emerged as a promising oncolytic platform that may not be compromised by pre-existing MeV immunity [86,87,88,89,90,91]. Preclinical virotherapeutic studies have been primarily performed using MeV vaccine strains over wild-type strains, due to the exceptional genetic stability and safety record of the vaccine strains [92]. The development of oncolytic virotherapy provides a new approach to cancer therapy by selectively targeting tumor cells using previously identified tumor markers on the tumor surface [93]. 

### 4.1. MeV Receptor Expression in Cancer Cells

**Figure 4:**
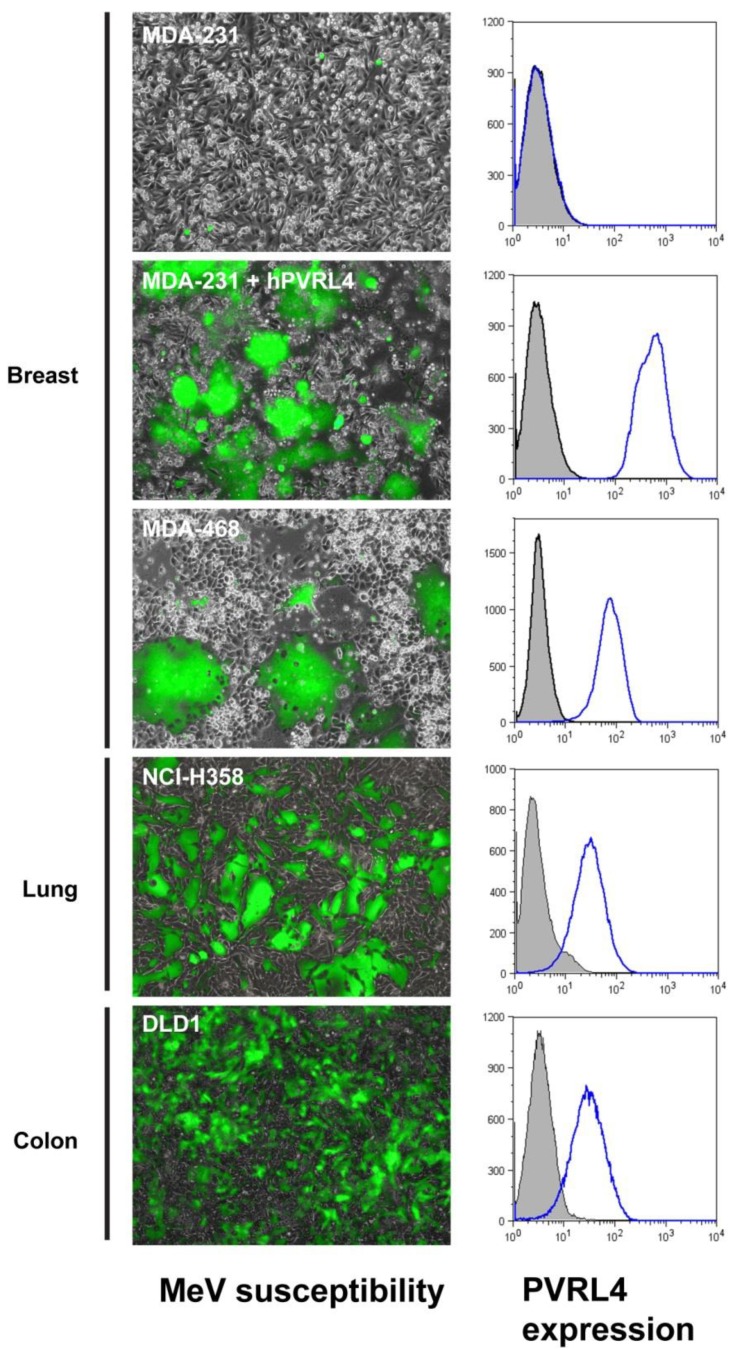
PVRL4 expression in breast ((MD M.D. Anderson (MDA)-231 + PVRL4, MDA-468), lung (National Cancer Institute (NCI-H358)) and colon ((D.L. Dexter (DLD-1) carcinoma cells correlates with wtMeV infection [21]. Adenocarcinoma cells were infected with wtMeV-GFP (Ichinose-323 strain, Roberto Cattaneo, Mayo Clinic) at a multiplicity of infection (MOI) of 0.1, and virus replication was monitored by microscopy at 72 h post infection. Cell lines were monitored for PVRL4 expression by flow cytometry using specific rabbit polyclonal antibodies. PVRL4-positive cells were susceptible to wtMeV infection.

Numerous studies have reported SLAM, CD46 and PVRL4 as being tumor cell markers. A number of previous clinical case studies documenting “spontaneous” tumor regression in patients with Hodgkin’s disease and Burkitt’s lymphoma were reported after concurrent measles virus infections [94, 95]. These tumors are known to express elevated levels of SLAM on their surface [96], suggesting that MeV infection may have been responsible for the apparent tumor regression. The oncolytic efficacy of MeV in T-cell lymphomas has also been observed [97, 98]. Secondly, CD46 is expressed at low levels on all nucleated cells [99] and is upregulated in numerous cancers (breast, cervical, colorectal, gastrointestinal, lung, leukemias and multiple myelomas) [100,101,102,103,104,105,106]. Attenuated vaccine strains of MeV can preferentially infect cancer cells expressing higher densities of CD46 molecules on their surface [107, 108]. Finally, PVRL4 is upregulated on the tumor surface of breast [109], lung [110], ovary [111] and colon [21]. During the process of tumorigenesis, expression of PVRL4 promotes the anchorage independence and cell-cell clustering in epithelial cells by engaging PVRL1 on juxtaposed cells [112]. Given the restricted PVRL4 distribution to most human body tissues compared to the ubiquitous CD46 expression and the presence of SLAM on activated lymphocytes and dendritic cells, PVRL4 is a promising tumor-associated marker for cancer therapy. MeV can successfully infect PVRL4-positive adenocarcinoma cells derived from lung, breast and colons tumors (Figure 4) [21]. 

### 4.2. Enhancing MeV Oncolytic Activity and Anti-Tumor Immunity

Genetic engineering is widely used to enhance MeV oncolytic activity and selectively target tumors [88]. A recombinant MeV that was unable to use SLAM (SLAM blind) targeted PVRL4 on human breast cancer xenografts in immunodeficient mice and showed oncolytic activity [113]. In addition to targeting tumor markers, other strategies increase oncolytic activity by arming oncolytic viruses with therapeutic genes [114,115,116] or using vesicular stomatitis virus (VSV)/MeV hybrid viruses as an oncolytic platform [117]. This hybrid incorporates the powerful replication machinery of VSV and encodes both MeV H and F instead of the VSV-G attachment protein [118]. The VSV/MeV hybrid virus is capable of enhanced spread and a cytopathic effect in cancer cell lines compared to MeV alone [118]. Unfortunately, the tumor fighting ability of such hybrid viruses is restricted by the high sensitivity of VSV to the type I IFN response [119, 120]. However, preliminary studies indicate that recombinant viruses incorporating the PVRL4 binding properties of MeV H could be useful in targeting and destroying PVRL4 positive tumors. 

**Figure 5:**
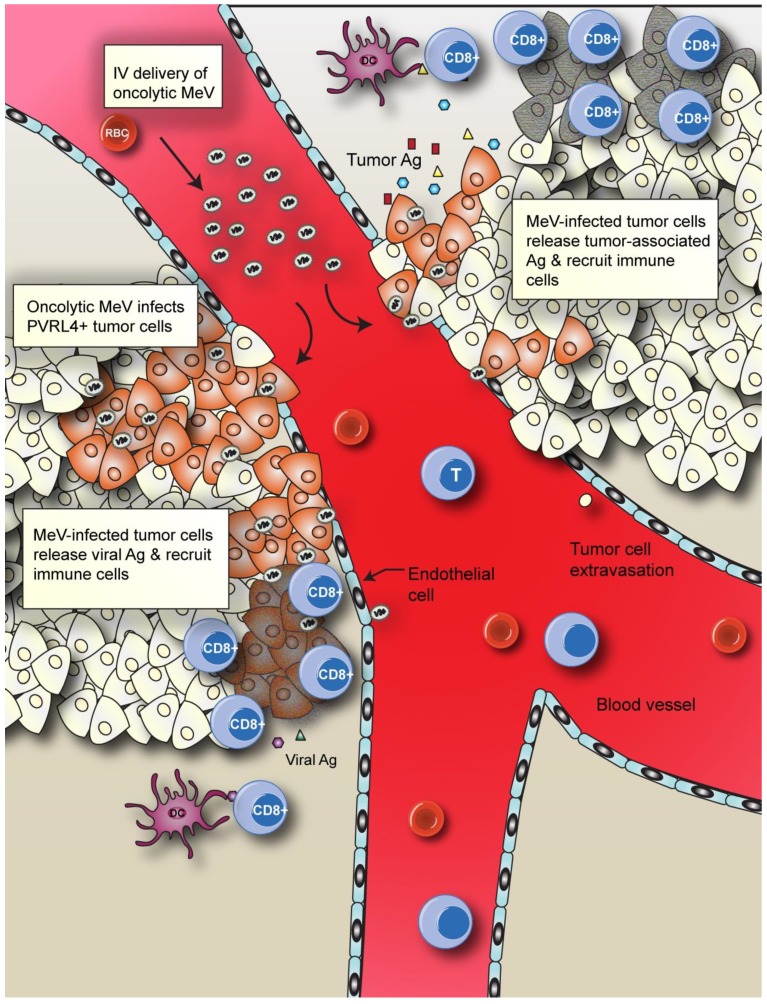
Oncolytic MeV induces both virus-mediated tumor cell lysis and immune cell recruitment to the tumor site. Intravenous (IV) delivery of oncolytic MeV crosses the endothelial cell layer and infects PVRL4 positive tumors. Infected cells (orange cells) are lysed and release tumor-associated antigens, which are recognized by immune cells, including dendritic cells (DC) and neutrophils. DCs present tumor peptides to T-cells (CD8+), which induces an anti-tumor immune response, leading to enhanced tumor cell killing (grey tumor cells) by the host immune system.

A major concern with oncolytic virotherapy, however, is how to continue tumor regression once the virus is cleared from the host. Inducing anti-tumor immunity may be one strategy to continue ongoing tumor destruction once the virus has been cleared (Figure 5). Other oncolytic viruses, like VSV, can induce anti-tumor immunity in models expressing exogenous antigens [121]. Several studies have investigated the role of anti-tumor immunity after MeV virotherapy. For example, activated neutrophils were found to be important for tumor regression following MeV oncolytic therapy. Infection of human lymphoma xenografts in mice with MeV that expressed the granulocyte macrophage colony stimulating factor (GMCSF) recruited neutrophils to the tumor site, resulting in enhanced tumor regression [122]. In addition, Guillerme *et al.* [123] observed that MeV-infected melanoma cell lines are efficiently internalized by plasmacytoid DCs, leading to the presentation of tumor-associated antigens to CD8+ T-lymphocytes and sustained tumor regression. Although intravenous administration of oncolytic viruses is the preferred method of delivery, the likelihood that virus particles will encounter neutralizing antibodies is increased [124]. A number of strategies are being developed, such as receptor switching, the use of cell carriers or immunosuppressive drugs, to overcome this potential hurdle [125].

Finding the right balance between tumor destruction by MeV and eliciting host anti-tumor immunity will be key considerations in eradicating tumors using oncolytic measles virotherapy.

## 5. Conclusions

Identification of PVRL4 as an epithelial cell receptor for MeV, CDV and PPRV is a major advance in the morbillivirus field. Indeed, this discovery provides a better understanding of morbillivirus pathogenicity and establishes a new paradigm for the spread of virus from lymphocytes to airway epithelial cells and virus shedding into the lumen of the lungs. Identification of inhibitors that block membrane fusion and entry by measles virus have been previously reported in the literature. However, the identification of important amino acid residues within the V domain structures of SLAM and PVRL4 could lead to the development of prophylactic antiviral agents that block virus attachment during the early (lymphocyte) and late (epithelial cell) stages of infections. Finally, many adenocarcinomas express PVRL4 on their cell surfaces, making them obvious targets for oncolytic and immune therapy based upon recombinant morbilliviruses.
